# What if parental love is conditional …? Children’s self-esteem profiles and their relationship with parental conditional regard and self-kindness

**DOI:** 10.1186/s40359-023-01380-3

**Published:** 2023-10-09

**Authors:** Malin Brueckmann, Ziwen Teuber, Jelena Hollmann, Elke Wild

**Affiliations:** 1 Department of Psychology, Bielefeld University, Universitaetsstraße 25, Bielefeld, 33615 Germany; 2 Behavioural and Cognitive Sciences, University of Luxembourg, 11, Porte des Sciences, Esch-sur-Alzette, L-4365 Luxembourg

**Keywords:** Parenting, Life satisfaction, Self-compassion, Self-judgement, Latent profile, And latent transition analysis

## Abstract

**Background:**

Numerous studies have demonstrated that low, unstable, or contingent self-esteem negatively affects youth development and is linked to adolescent psychopathology. However, most previous studies have applied variable-oriented approaches, and less is known about the natural combination of self-esteem facets in school-aged adolescents, how parental conditional regard affects self-esteem profiles, and how these profiles relate to self-kindness, self-judgement, and life satisfaction.

**Methods:**

By employing a longitudinal person-oriented approach (i.e., latent profile analysis and latent transition analysis) on two-wave longitudinal data from 587 German secondary school students (52.3% female, *M*_age_=13.52 years), this study aims to (1) identify adolescents’ self-esteem profiles based on the level, stability, and contingency of self-esteem; (2) examine the impact of parental conditional regard on the self-esteem profiles explained using self-determination theory; and (3) examine these profiles’ relationship with self-kindness, self-judgement, and life satisfaction.

**Results:**

Four self-esteem profiles were derived: *optimal-secure* (~ 8%), *good* (~ 18%), *average* (~ 36%), and *low-insecure* (~ 38%). The results reveal a concerningly high proportion as well as a high stability of *low-insecure* self-esteem (~ 98%) and indicate the strong negative influence of parental conditional regard on the development of *optimal-secure* self-esteem. Furthermore, the results demonstrate strong correlations between *optimal-secure* self-esteem, highly developed self-kindness, and high life satisfaction.

**Conclusions:**

Using a longitudinal person-oriented approach, it was possible to identify a group with highly vulnerable self-esteem, characterised by particularly low self-kindness, strong self-judgment, and lower life satisfaction. The findings of this study support the need for prevention and intervention targeting adolescents with *low-insecure* self-esteem.

**Supplementary Information:**

The online version contains supplementary material available at 10.1186/s40359-023-01380-3.

## Introduction

Self-esteem (SE) is a central issue in the aetiology of various clinical disorders and thus requires significant attention in the context of therapeutic interventions [1, 2]. For example, in research on the development of depression, there is substantial empirical support for the vulnerability model, in which low SE is assumed to be the central vulnerability factor in the emergence of rumination and the development of depression [3, 4]. On the other hand, positive self-image acts as a resilience factor and weakens the link between critical life events and depression [5]. Although the complexity of aetiological models should not be underestimated, it is not only the development of depression that can be traced back to dysfunctional self-image and the individual’s own value attributions: SE also plays a central role in the development of personality and eating disorders, as well as addictive disorders (to name just a few examples; [1, 6]). SE manifests itself as early as childhood and adolescence [7, 8], during which parents are the most important caregivers for their children and serve as a significant source of resonance. Their love and appreciation, which are not conditional on their children’s appearance, performance, or behaviour, contribute to the development of optimal, secure SE and shape the child’s compassion towards themselves [9]. Such unconditional positive regard was already emphasised by Carl Rogers [10] within the framework of client-centred psychotherapy in order to provide clients with positive relational experiences and thus guide them to value and treat themselves kindly, regardless of their performance.

It is not surprising, then, that SE as a central vulnerability or resilience factor is one of the most researched topics in the behavioural and social sciences and is increasingly being considered alongside conditional regard, self-kindness, and self-judgement [11, 12]. To date, however, many studies have followed a nomothetic, variable-oriented approach and considered the SE facets separately. This approach has resulted in many contradictory findings, especially with regard to optimal SE (see [13, 14]). To our knowledge, there is a lack of idiographic, person-oriented approaches to studying SE in childhood and early adolescence. Since these stages are central to the development and stabilisation of SE and personality and their effects persist across the lifespan (see [15, 16]), a holistic view of SE profiles and their relationship with parental academic conditional regard (PACR), self-kindness, self-judgement, and life satisfaction is particularly important in these age spans. Not least, this holistic view helps to clarify what optimal SE looks like, how it is influenced by PACR, and whether an optimal, secure SE is a sign of a healthy, resilient personality. To this end, we conducted a two-wave study of 587 school-aged adolescents and applied latent profile and transition analyses to identify SE profiles. We then explored these profiles’ stability and tested their relationship with possible predictors (PACR) and outcomes (self-kindness, self-judgement, and life satisfaction).

### Global self-esteem and self-esteem facets

Global SE denotes an individual’s subjective evaluation and assessment of themselves and thus represents their attitude toward their own person [17–19]. However, in current research, global SE has long since ceased to be considered; instead, the differentiated examination of individual facets (level, stability, and contingency) and their interactions has increasingly come to the fore.

Level of SE refers to the extent to which the individual (de)values and (dis)likes their own person. A person’s SE can therefore be positive and high or negative and low. For example, high global SE is demonstrated to be positively related to life satisfaction [20], work engagement, and academic success [21] and has been identified as a protective factor against psychological problems in general [22]. In contrast, lower global SE is associated with more mental health problems such as anxiety and depression [23], procrastination [24], and social difficulties [25]. However, there are also contradictory findings regarding whether high SE is exclusively beneficial [11, 13], prompting more attention to be paid to additional SE facets.

The stability of SE is one of the facets that has been used to elucidate contradictory results regarding optimal SE (cf., [26]). The extent of changes in SE experienced over time, as well as the subjective assessment of the fragility and robustness of the SE level, are subsumed under the facet of SE stability (cf., [27]). Individuals with unstable compared to stable SE are characterised by stronger affective swings and higher reactivity in behaviour [28]. However, overall, SE stability alone does not have significant predictive power, so it is often examined in combination with SE level. Regarding the interaction between the SE facets of level and stability, low and stable SE is more likely to lead to depression [29], whereas high but unstable SE is associated with reactive positive affects following receipt of positive feedback but equally with strong defensive and devaluing tendencies following receipt of critical feedback [2, 30, 31].

Thus, individuals differ in the extent to which the level of their SE depends on external factors such as the achievement of self-imposed goals and standards or those defined by relevant others (SE contingency; [28, 32, 33]). High SE contingency denotes a strong reliance on positive external feedback, which arises because the individual does not consider the self to be intrinsically valuable but ties their worth to success and effort. Consequently, it is not surprising that Otterpohl et al. ([32] p. 988) state, ‘Research in the past decades found that CSE [contingent self-esteem] is associated with several negative outcomes, often even above and beyond any effect of global self-esteem (e.g., financial, social, and academic problems, depression, drinking, and anger)’. SE contingency not only contributes to the elucidation of contradictory findings when combined with the other SE facets but also, when considered on its own, is related to life satisfaction and well-being [34–37], parenting [37, 38], and self-compassion [39].

### Self-esteem profiles

If we consider possible compositions of the different facets, several different profiles can be assumed to exist. First, there is a high, stable, and less contingent SE profile. This SE profile is understood to be synonymous with true SE, which is independent of external conditions and feedback and is considered to be less fragile (optimal-secure SE; [28, 40, 41]). It does not have to be tested but arises without continuous self-evaluation [42, 43]. Furthermore, it is considered to be a resource, associated with particularly favourable development and reduced risk of mental health problems [44]. In contrast, and consistent with previous research, a second expected SE profile is an insecure one that is unstable and highly contingent. This expectation is in line with Kernis’ [28] postulation of an insecure SE, which is characterised by high vulnerability to psychological disorders. Because of the highly contingent nature of this SE profile, children and adolescents with such a profile strive to improve themselves through effort, achievement, and success [12, 45]. However, an insecure SE profile may be associated with either low or with high SE levels. It is possible that the level of SE in this context depends primarily on successes, for example academic performance, and thus on the activation of negative or positive self-attributions. In the context of narcissism, scholars have applied variable-oriented approaches to analyse discrepancies in explicit and implicit SE, obtaining results that suggest fragile (explicitly high and implicitly low SE levels; e.g., [46, 47]) or damaged SE (the other way around; e.g., [48, 49]). These results further emphasises that insecure SE can occur with both high and low SE levels. However, an insecure SE profile is tied to increased risk of frustration, excessive demands, and failure in relation to the individual’s own performance aspirations [50]. This risk results in a third SE profile that can be described in terms of learned helplessness [51] as low, stable, and less contingent. Children and adolescents who notice that they do not achieve the desired successes and do not receive positive feedback despite their efforts may give up on themselves [52, 53]. Lastly, a fourth profile, in which all three facets are average and undifferentiated, would also be conceivable and in line with previous research [54].

Despite the aforementioned considerations regarding qualitatively different SE profiles, identifications of the number and composition of the profiles are exploratory, as there is little evidence regarding the holistic view of the three SE facets [54], and current findings on the individual SE facets are inconclusive.

#### Stability of self-esteem porofiles

Based on the definition of global SE as a general, enduring tendency to assess the value of one’s own person [55], it can be assumed that the SE profile structures and profile memberships are quite stable over the lifespan. This assumption is supported by the rank-order stability of SE (estimated. 64; [56]), which is comparable to the rank-order stability of basal personality traits [17]. If changes in profile memberships arise, the general tendency in assessing SE should remain stable, so that individuals could be assigned to similar or related profiles over a shorter or longer time period (e.g., changes from an optimal-secure to an insecure SE profile seem unlikely and would counteract the formation of a general, enduring tendency to feel either valuable or worthless).

#### Self-esteem profiles and parental academic conditional regard

Various prominent theories regarding the development of SE (e.g., the *internalisation of early social experiences model*, *attachment theory*, *symbolic interactionism theory*, and *sociometer theory*; for an overview, [11]) share the view that parental behaviour may be the most important factor influencing children’s self-representation. In the last decade, parental conditional regard has been identified as a central and frequently used parenting strategy, especially in the academic domain, that frustrates basic psychological needs, as suggested by *self-determination theory* (SDT; e.g., by creating an inner ambivalence between autonomy and relatedness). It is considered highly autonomy-suppressive and thus harmful to child (SE) development [40, 57, 58]. PACR is understood as a specific type of controlling parenting behaviour where parental appreciation is dependent on the child meeting the parent’s expectations in the academic domain. Thus, children may try to retain their parents’ affection by behaving as desired and striving to meet their parents’ expectations [59]. Here, PACR includes not only withdrawal of love in response to undesirable child behaviour (parental academic negative conditional regard, PANCR) but also increases in parental appreciation in response to the child meeting parental education standards (parental academic positive conditional regard, PAPCR; [60]). It has been shown that not only PANCR but also PAPCR are associated with strong disadvantages (e.g., emotional and motivational costs) compared to autonomy-supportive parenting strategies, while the effects of PANCR are generally stronger than those of PAPCR [61]. In any case, PACR – both PAPCR and PANCR – does not lead to children developing optimal, secure SE in the long run.

From the SDT perspective, PACR frustrates the satisfaction of the basal psychological needs for autonomy, experience of competence, and belonging, which are assumed to be essential for personal growth, well-being, and integrity [15]. PACR has a strong autonomy-suppressing effect; children and adolescents must behave in a certain way to receive affection and appreciation from their parents (satisfaction of the need for belonging), which is why they increasingly act on the basis of external pressure, shame, and guilt rather than their own intrinsic motivation [15, 62]. This results in introjected action regulation, and, in the long run, children begin to integrate the (academic) conditions for self-appreciation into their value system and perceive external pressures as their own internal pressures [41, 63].

In recent years, the positive connection between SE contingency and PACR has been repeatedly demonstrated [60, 64–66]. In addition, children with parents high in PACR show lower SE levels [67] and lower SE stability, which vary along with academic outcomes [68]. While PAPCR is primarily associated with high SE contingency [68, 69], PANCR has an additional negative effect on the level of SE [67, 68]. Withdrawal of parental love and attention in the event of the child’s academic failure is likely to pose a direct threat to the child’s SE and, following SDT, to thwart the child’s ability to experience competence. Since PAPCR and PANCR cannot be considered two sides of the same coin [12], it is justifiable to consider their relationships with the different SE profiles separately.

Based on the above, it can be assumed that more PACR in general leads to a more contingent and possibly less stable (more insecure) SE. Regardless of SE level, insecure SE is often accompanied by avoidance of mistakes and hiding of one’s own weaknesses (e.g., [12]), since children try to avoid direct or indirect negative parental feedback on their performance. Furthermore, as the insecurity of SE increases, so does the tendency to devalue oneself on the basis of negative performance. The use of PANCR in particular is expected to be associated with a low, unstable, and highly contingent SE profile. In contrast, a high, unstable, and highly contingent SE profile might be more related to the use of PAPCR. This is because children with this profile can be described as highly engaged and motivated, which can contribute to success and meeting expectations, at least in the short term [12]. If children of parents who show PACR (and especially PANCR) consistently fail to meet parental demands despite high effort expenditure, learned helplessness is likely to occur, and low, stable, and low-contingent SE develops. For the average SE profile (where all three facets are in the average range), we assume less PACR than for the two profiles already mentioned, and we expect the development of optimal, secure SE to be associated with the lowest amount of PACR.

#### Self-esteem profiles, self-kindness, self-judgement, and life satisfaction

Optimal, secure SE (high, stable, and less contingent) allows children and adolescents to integrate their own strengths, weaknesses, and failures because they are not seen as threatening to their self-worth [28, 44]. This enables them to treat themselves with respect, acceptance, and friendliness, regardless of their performance, and not to devalue themselves on the basis of mistakes. It is precisely this positive, accepting, and tolerant attitude towards oneself even when considering one’s own weaknesses and imperfections that Neff [70] describes as self-compassion. In addition to the facets of mindfulness versus overidentification and connective humanity versus isolation, self-kindness and a low tendency towards self-judgement are central aspects of self-compassion and include this patient and benevolent, rather than punitive and judgemental, attitude towards the self [70].

Following the ‘self-compassion-as-a-consequence model’, in which ‘believing you are a person of worth […] is a precursor to feeling worthy of SC [self-compassion]’ ([71] p. 620), we assume that optimal, secure SE correlates with more self-kindness and less self-judgement. The opposite is expected for insecure SE (regardless of SE level), as high contingency leads to devaluation of the self in the face of imperfections. Furthermore, for the profile of learned helplessness (low, stable, and less contingent), high self-judgement and low self-kindness are assumed, since the person considers themselves to be completely failed and worthless anyway. For the average SE profile, we expect values somewhere in between: that is, more self-kindness and less self-judgement than for the insecure and learned helplessness profiles, respectively, and less self-kindness and more self-judgement than for the optimal, secure SE profile.

Over the last few decades, it has been demonstrated that life satisfaction is significantly related to mental health (for an overview, see [72]) and that SE is one of the central predictors of both life satisfaction and mental health [20]. Insecure SE varies depending on external circumstances [35] and leads to constant tension and strain due to high performance pressure. In turn, this results in lower life satisfaction and a higher risk for psychological disorders (e.g., depression; [69]). Therefore, the highest life satisfaction is expected to co-occur with optimal, secure SE and to decrease across the following order of profiles: average SE, insecure SE (lower life satisfaction with reduced SE level), and learned helplessness.

## Current study

### Research objectives

As one of the first, the present study aims to identify SE profiles in a sample of secondary school students to examine the six-week stability of profile membership as well as the relationship between SE profile and PACR, self-kindness, self-judgement, and life satisfaction. For this purpose, a longitudinal, person-oriented approach was adopted to address three research objectives.

The first main research objective was to identify the number and characteristics of SE profiles with respect to the three facets of level, stability, and contingency. We expected to find at least four profiles: (P1) optimal-secure (high, stable, less contingent), (P2) average (all three facets in the average range), (P3) insecure (either with low or high SE levels or both profiles), and (P4) learned helplessness (low, stable, less contingent). Furthermore, we explored the stability of the profiles: we expected the affiliation to individual profiles to be relatively stable over a six-week period (as well as over longer time spans), and any occurring transitions to be made only to related profiles.

The second research objective was to investigate the relationship between PACR and the probability of membership of the different SE profiles. We expected that both more PAPCR and PANCR would predict the development of the insecure (P3) and learned helplessness (P4) SE profiles, while the optimal-secure profile [1] would be associated with the lowest expressions of PACR.

The third research objective was to examine possible relationships between SE profile membership and self-kindness, self-judgement, and life satisfaction. For students with optimal-secure SE (P1), we expected more self-kindness, less self-judgement, and more life satisfaction. We hypothesised that students with the average (P2), insecure (P3), and learned helplessness (P4) SE profiles would show more self-judgement as well as less self-kindness and life satisfaction in the corresponding order.

### Covariates

To investigate the relationships between SE profiles, PACR, self-kindness, self-judgement, and life satisfaction, it is important to consider possible time-invariant covariates. In particular, previous research has identified gender, socioeconomic status (SES), and migration background as significant influencing factors in relation to parenting behaviour, SE, self-kindness, self-judgement, and life satisfaction. For example, SES is positively related to less controlling parenting strategies [73, 74] as well as adolescents’ mental health [75] and SE [76]. For gender, inconsistent findings emerge with respect to SE, suggesting that, to the extent that significant differences occur, boys have more positive and stable SE than girls. In relation to migration status, for example, lower life satisfaction [77] and lower global SE [78] are evident. To avoid confounds of gender, SES, and migration background, these factors were integrated as covariates in the analyses.

## Method

### Participants and procedure

The original sample in the current study consisted of 727 students (mean age at T1 = 13.55 years, *SD =* 1.09, age span from T1 to T2 = 12–18 years) from German schools of different types (including academic and non-academic school tracks). The data were collected between March and June 2017 at two measurement time points with an average interval of six weeks. Participation in the survey was voluntary; all parents and students were required to complete an informed consent form beforehand. Approval was obtained from the Ethics Committee of Bielefeld University and all methods were carried out in accordance with relevant guidelines and regulations. The survey was administered on site during one hour of the school day and was guided by two trained instructors.

After removing students who either participated at only one measurement time point or had missing values on over half of the scales of the relevant constructs, data for 587 students (52.3% female) remained in the analysis. In this sample, 205 participants had a migration background, 40% were in seventh grade, 44.9% in eighth grade, and 15.1% in ninth grade.

#### Missing data analysis

For the analysis of missing values, the data from the original sample of 727 students (excluding 7 students who participated at T2 but not at T1, e.g., due to illness) was compared with the T1 data from students who dropped out at T2 (104 students). Another 29 students had more than 50% missing values on the relevant scales and were therefore excluded from further analyses. Hence, a total of 616 students participated at T1 and did not drop out until T2 (51.2% girls, 36.1% with a migration background), and a total of 587 reliable pairs of data remained for further analyses. In our study, boys had a significantly higher probability of dropping out (*χ*^*2*^ = 12.135, *p* = .002, Cramer’s *V* = 0.129; [79, 80]). Furthermore, adolescents who dropped out reported higher levels of experienced PAPCR (*t* = 2.216, *p* = .028, *d* = 0.242) and significantly lower life satisfaction (*t* = − 2.847, *p* = .005, *d* = 0.337). No significant effects were found for SES (*χ*^*2*^ = 1.01, *p* = .908), migration background (*χ*^*2*^ = 0.093, *p* = .345), PANCR (*t* = 0.375, *p* = .708), self-kindness (*t* = − 1.582, *p* = .116), or self-judgement (*t* = 1.225, *p* = .223), nor for level of SE (*t* = − 1.679, *p* = .094), SE stability (*t* = – 0.125, *p* = .901), or SE contingency (*t* = 0.021, *p* = .983). In further analyses, we used the robust maximum likelihood estimator to handle missing values. Gender, SES, and migration background were included as covariates.

### Measures

Predictors (PAPCR and PANCR), covariates (gender, migration background, and SES), and outcomes (self-kindness, self-judgement, and life satisfaction) were measured as manifest variables (i.e., scale mean measured at T1 and T2). The four SE profiles are composed of three facets: level, stability, and contingency of SE. These corresponding facets were modelled as latent variables with multiple indicators measured at both time points. In what follows, we report McDonald’s omega as a measure of internal consistency because it reflects the proportion of variance in the scale results that is explained by the overall latent factor [81, 82].

#### Facets of self-esteem

The three SE facets were assessed using the *German* *Self-Esteem Inventory for Children and Adolescents* [33] and rated on a five-point Likert scale (1 = *does not apply*, 5 = *applies*). The original scale of 30 items was shortened to 12 items by integrating the items with the highest loadings on the respective factors (following the manual) into the short version. Thus, all three facets included four items each. The scale for level of SE showed good internal consistency (e.g., ‘I feel worthy’; ω_T1_ = 0.823, ω_T2_ = 0.846). The items of the SE stability scale (e.g., ‘Whether I feel good or not actually changes all the time’; ω_T1_ = 0.798, ω_T2_ = 0.836) were recoded so that higher scores represent higher stability. An example item on the SE contingency scale is ‘I feel more valuable somehow when I get good grades’; ω_T1_ = 0.720, ω_T2_ = 0.797). According to the results of the confirmatory factor analysis (CFA), the three-factor structure could be found in our data.

#### Parental academic conditional regard

The two facets of PACR – PAPCR and PANCR – were measured using nine items, each adapted from the *German Parental Academic Conditional Regard Inventory* [15, 83]. This instrument records parental affective and behavioural reactions in terms of increased (PAPCR) or withdrawn attention (PANCR) based on children’s school performance. An example for PAPCR is ‘When I get good grades in school, I notice that my main caregiver praises me by paying more attention to me’ (ω_T1_ = 0.939, ω_T2_ = 0.949). The PANCR scale (e.g., ‘When I get a bad grade in school, I realise that my primary caregiver is punishing me with disrespect’) also showed excellent internal consistency at both measurement points (ω_T1_ = 0.922, ω_T2_ = 0.950). All items were rated on a five-point Likert scale (1 = *not true at all*, 5 = *is exactly right*). The solution of the two-factor CFA fit the data well.

#### Self-compassion

Self-kindness and self-judgement were considered as the two essential facets of self-compassion and measured via the *Self-Compassion Scale* [70, 84]. Both scales included five items, which were rated on a five-point Likert scale (1 = *very rare*, 5 = *very often*). An example of a self-kindness item is ‘I try to be understanding and patient towards those traits of my personality that I don’t like’ (ω_T1_ = 0.726, ω_T2_ = 0.781). In comparison, ‘I disapprove of and condemn my own mistakes and weaknesses’ is an example of a self-judgement item (ω_T1_ = .740, ω_T2_ = .776). The two-factor structure was approved in our data.

#### Life satisfaction

The *Satisfaction With Life Scale* (SWLS; [85, 86]) was used to measure one aspect of adolescents’ well-being. It consists of five items (e.g., ‘I am satisfied with my life’; ω_T1_ = 0.824, ω_T2_ = 0.843) that are scored on a five-point Likert scale (1 = *does not apply*, 5 = *applies*). This scale was also unidimensional in our study.

#### Covariates

Adolescents’ gender, migration background, and SES were considered as covariates. Three response categories were provided for gender (1 = *male*, 2 = *female*, 3 = *diverse*). Migration background was dummy coded as 0 = *no migration background* and 1 = *migration background* if at least one of the respondent’s parents or grandparents had been born abroad. Respondents’ SES was assessed by the number of books available in the household using a five-point response scale ranging from 1 = *0–10 books* to 5 = *over 200 books* [87].

### Analysis strategy

For the data analysis, the Morin and Litalien [88] teaching paper was used for guidance. Latent profile analysis (LPA) and latent transition analysis (LTA) were performed to estimate the SE profiles and the transitions between these profiles within the six-week time span. All data analyses were conducted in *M*plus 8.6 [89].

#### Longitudinal measurement invariance

Before investigating our research questions, we tested the longitudinal measurement invariance of the SE facets. Once the final model of measurement invariance had been established, the corresponding factor scores (estimated in standardised units as *M* = 0, *SD* = 1) of the SE facets were saved for further analyses. Compared with Z-scores, which are standardised scores representing how far each data point is from the mean in standard deviation units, factor scores provide a more direct representation of the latent constructs and have the key advantage of partially controlling for measurement error while maintaining the hierarchical nature of the measurement model [90].

#### Latent profile analysis

To determine the number of SE profiles (Research Objective 1), cross-sectional LPA models were set up separately for both measurement time points. This decision was based on both theory and consideration of statistical characteristics. For statistical adequacy, the following criteria were taken into account: Akaike’s information criterion (AIC), the Consistent AIC (CAIC), the Bayesian information criterion (BIC), the Adjusted BIC (ABIC), entropy, and the Lo-Mendell-Rubin likelihood ratio test (LMR-LRT). The smaller the AIC, CAIC, BIC, and ABIC, the better the fit of the model. Furthermore, significant (*p* < .05) LMR-LRT values indicate that a model with *k* profiles fits the data better than a model with *k*-1 profiles, whereas higher entropy values (ranging from 0 to 1) reflect higher classification accuracy.

#### Latent transition analysis

Following the identification and determination of the number of profiles for each of the measurement time points, we integrated the cross-sectional LPA models into a longitudinal LPA model and tested for profile similarity by following four steps (see [91]). In the first step, we investigated the configural similarity to test whether, at each measurement point, the same number of profiles could be identified using the same indicators. Next, we held the mean values of the profile indicators constant to check the structural similarity of the profiles longitudinally. If both configural and structural similarity (as the prerequisite for all further steps) were obtained, we performed a test of dispersion similarity. Here, we examined the extent to which differences within profiles were similar across measurement time points by holding the indicator variances constant over time. Finally, we tested distributional similarity to determine whether the probability of profile membership differed over time. To identify the most similar model, we employed the fit indices of CAIC, BIC, and ABIC. The rule is that at least two of the indices should decrease with the addition of further restrictions [88, 92]. To test the stability of profile affiliations and the transitions between profiles over the six-week period (Research Objective 1), the model identified as most similar was transferred into an LTA model, an extension of LPA using longitudinal data. We followed the manual auxiliary three-step approach [93].

Subsequently, it was possible to identify the associations between profile membership and covariates, predictors, and outcomes by including them in the LTA model from the previous step (Research Objectives 2 and 3). To test the effects of presumed covariates and predictors on profile membership (predictive similarity) over time, each of the three covariates (i.e., gender, migration background, and SES) and the two parental conditional regard factors (i.e., PANCR and PAPCR) were included as predictors in the LTA model. To examine whether the association between profile membership and outcomes persisted over the two measurement time points (explanatory similarity), each of the three outcomes (i.e., self-kindness, self-judgement, and life satisfaction) was added to the LTA model while controlling for all covariates and predictors.

## Results

The scale means and zero-order intercorrelations between the variables are shown in Appendix (1) The results regarding the measurement invariance of the SE facets are presented in Appendix (2) Based on these results, strong measurement invariance could be assumed, enabling the factor scores of the SE facets to be used in further analyses.

### Latent profiles of self-esteem facets

Regarding Research Objective 1, at T1, a four-profile solution was shown to be optimal (see Appendix 1). At T2, the LMR-LRT test was not significant for four compared to three profiles, but all four fit indices pointed to the four-profile solution. The corresponding fit indices also indicated that a five- or six-profile solution was more appropriate but produced profiles that comprised less than 5% of the participants that emerged from the five-profile solution (cf., [94]). Since the additional profiles were relatively close to the profiles produced by prior solutions and did not yield meaningful new insights regarding qualitative differences between profiles (i.e., they showed only minor differences in all three SE facets), they were not considered on the basis of parsimony (e.g., [95]). Accordingly, when the theoretical considerations, fit indices, and explanatory power of additional profiles were considered together, the four-profile solution was assumed to be the best fit for both measurement time points and showed higher classification accuracy (> 0.78) than solutions with more than four profiles (see Appendix 3). The next step in the analysis was to check the similarity of the four-profile solutions across the two measurement time points. As Appendix 4 shows, declining fit indices despite further restrictions supported the distributional similarity model, which formed the basis for all further analyses.

**Fig. 1:**
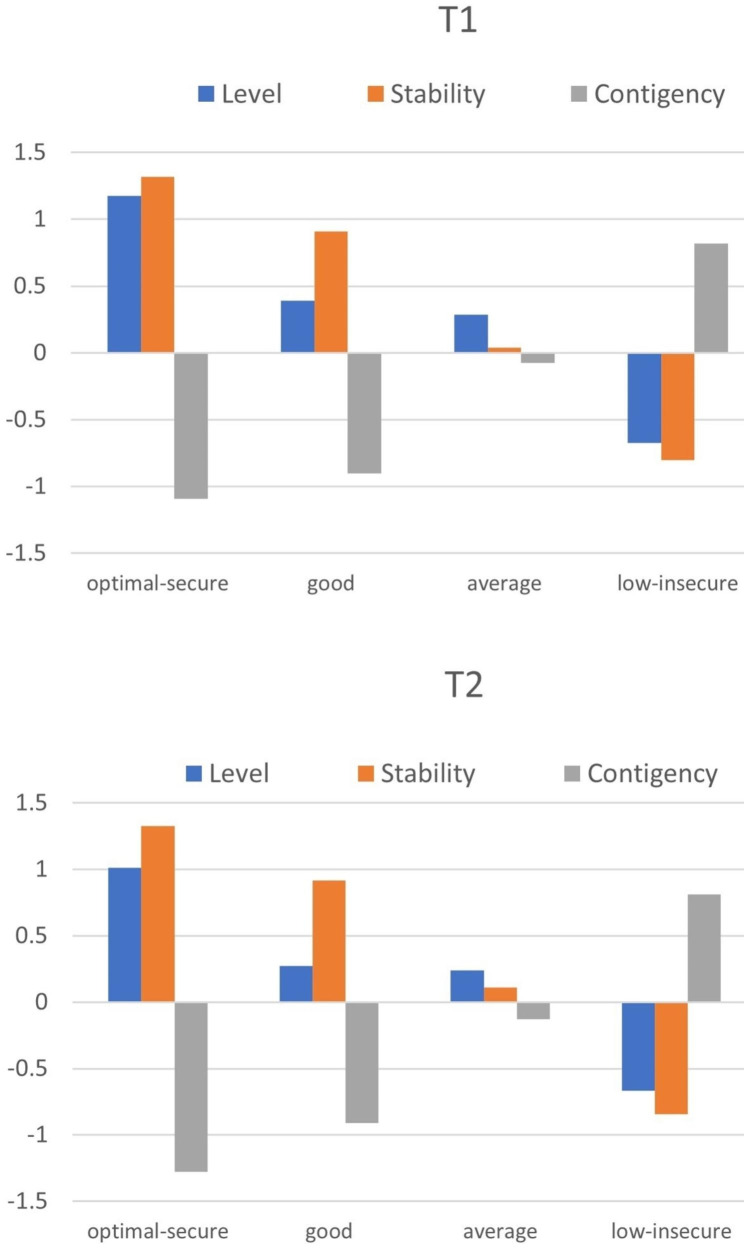
Self-esteem profiles at T1 and T2 based on distributional similarity model. Note. Level = self-esteem level; Stability = self-esteem stability; Contingency = self-esteem contingency

The first profile was labelled *optimal*-*secure* SE because students in this profile (6.80% at T1 and 4.94% at T2) had high values in terms of SE level and stability and extremely low SE contingency. The second profile had a similar pattern to that of the *optimal-secure* SE, with a slightly lower level of stability and slightly higher contingency. It was labelled *good* SE (21.30% at T1 and 13.28% at T2). Adolescents in profile 3 showed values close to 0 for the three SE facets, representing the *average* SE profile (35.61% at T1 and 40.38% at T2). Participants in the last profile reported low SE level and stability but very high SE contingency. Therefore, the last profile represented insecure SE with a low SE level and was thus called *low-insecure* SE (36.29% at T1 and 41.40% at T2). No further SE profiles, for example insecure SE with a high SE level or learned helplessness, were found when considering five- or six-profile solutions. Figure 1 provides an overview of the identified profiles.

#### Latent transitions between self-esteem profiles

**Fig. 2:**
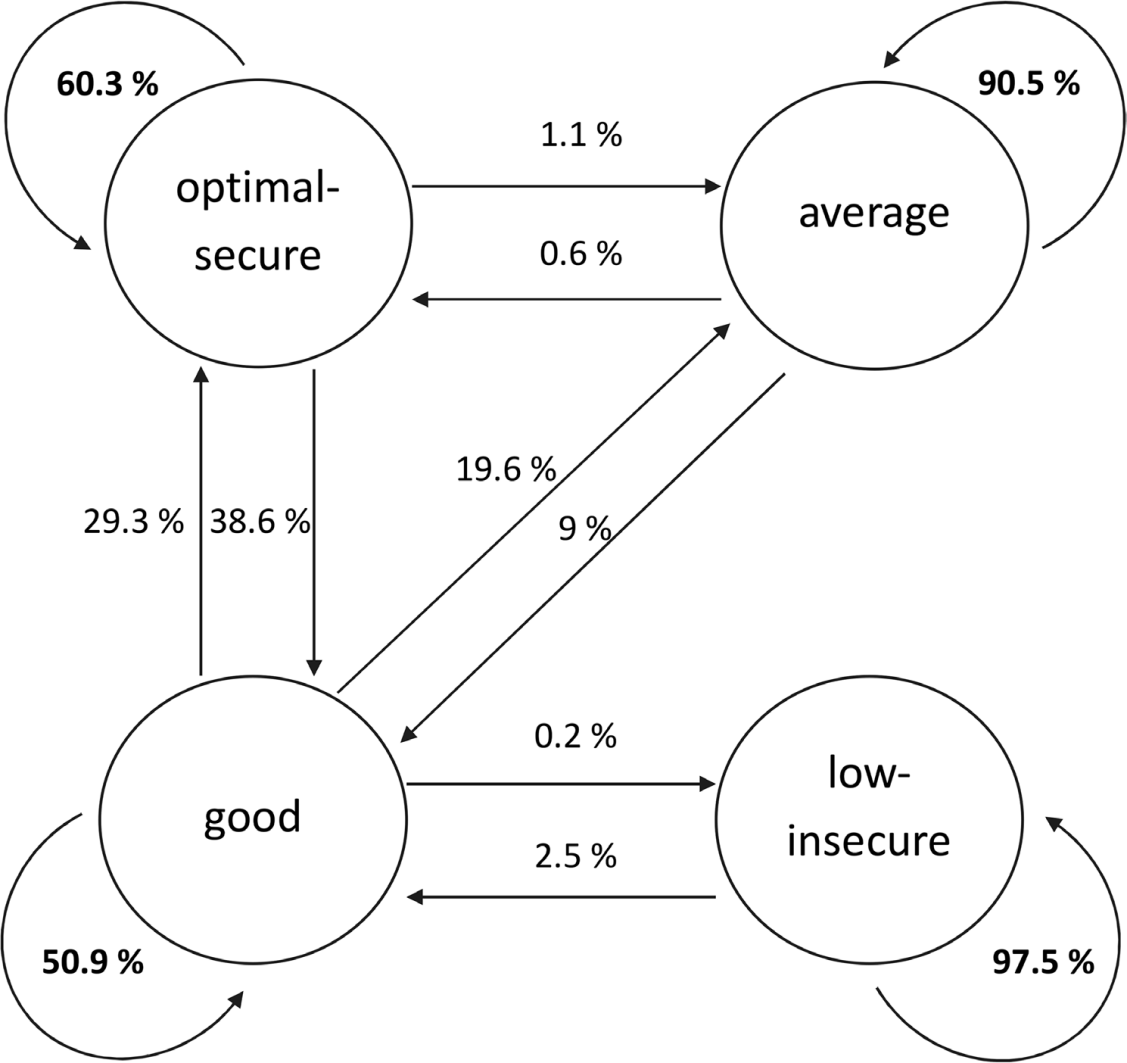
Transition probabilities (in %) of self-esteem profiles over time. Note. Stability estimates are in boldface. Transition probabilities sum up to 100% for each measurement occasion

In the next step (Research Objective 1), following the manual auxiliary three-step approach, we converted the distributional similarity model into an LTA model. In this LTA model, we examined the stability and change of the SE profiles over time (transition probabilities from T1 to T2 are summarised in Fig. 2). Overall, profile stability was high for all four SE profiles (particularly for the *low-insecure* profile). Transitions occurred almost exclusively to related profiles, consistent with our hypothesis.

#### Self-esteem profiles, predictors, and outcomes

We ran predictive similarity models based on the distributional LTA model to address the effects of covariates and PACR on SE profile membership. Multinomial logistic regression estimations are shown in Table 1. Boys consistently showed a significantly higher probability of belonging to an *optimal-secure* SE profile than girls (e.g., OR = 2.956, *p* < .01 for *low-insecure* vs. *optimal-secure*). Migration background was not significantly associated with the tendency to belong to an *optimal-secure* or *low-insecure* SE profile. In addition, SES was not a significant predictor of the development of SE profiles. For PACR – both PAPCR and PANCR – significant effects were obtained: in general, increased PACR raised the probability of belonging to the *low-insecure* compared to the *optimal-secure* SE profile (e.g., PANCR: OR = 8.016, *p* < .01).

**Table 1:** Results from multinomial logistic regressions for effects of predictors and demographics on profile memberships

	good *vs.* optimal-secure		average *vs.* optimal-secure		low-insecure *vs.* optimal-secure	
	β (SE)	OR	β (SE)	OR	β (SE)	OR
Gender	0.215 (.309)	1.240	0.506 (.302)	1.658+	1.084 (.326)*	2.956**
Migration	0.064 (.290)	1.066	-0.072 (.290)	0.931	0.084 (.302)	1.088
SES	0.079 (.118)	1.082	0.054 (.121)	1.055	0.021 (.127)	1.021
PANCR	0.534 (.886)	1.690	1.480 (.780)+	4.392+	2.081 (.797)**	8.016**
PAPCR	-0.032 (.165)	.968	0.328 (.149)*	1.388*	0.444 (.159)**	1.559**
	**good vs. low-insecure**		**average vs. low-insecure**		**good vs. average**	
	**β (SE)**	**OR**	**β (SE)**	**OR**	**β (SE)**	**OR**
Gender	-0.868 (.236)**	.420*	-0.578 (.217)**	.561**	− .56(.27)*	.748
Migration	-0.020 (.230)	.980	-0.156 (.228)	.855	0.136 (.235)	1.145
SES	0.058 (.090)	1.059	0.032 (.088)	1.033	0.025 (.089)	1.026
PANCR	-1.557 (.427)**	.211**	-0.602 (.180)*	.548**	-0.955 (.459)*	.385**
PAPCR	-0.476 (.122)**	.621**	-0.116 (.111)	.890	-0.360 (.129)*	.698**

*Note.* SE: Standard Error of the coefficient; OR: Odds Ratio; gender: 1 = male, 2 = female; migration: 0 = without migration background,

1 = with migration background; SES = socioeconomic status; PAPCR = parental academic positive conditional regard;

PANCR = parental academic negative conditional regard

* *p* < .05; ** *p* < .01. + *p* < .10

We ran explanatory similarity models based on the distributional LTA model to examine the influence of adolescents’ SE on their well-being and self-compassion. Table 2 presents the results for the SE profiles and outcomes (self-kindness, self-judgement, and life satisfaction) while controlling for the covariates and predictors. Students belonging to the *optimal-secure*, *good*, and *average* SE profiles showed significantly more self-kindness than members of the *low-insecure* profile. For the profiles *optimal-secure*, *good*, *average*, and *low-insecure*, in the corresponding order, significantly increasing mean values are shown with respect to self-judgemental thoughts and behaviours. In terms of life satisfaction, there was no significant difference between the *good* and *average* profiles, while there was significantly higher life satisfaction for the *optimal-secure* profile and significantly lower life satisfaction for the *low-insecure* profile.

**Table 2:** Associations between Profile Membership and Outcomes after Controlling for Covariates

	P1: optimal-secure *M* [CI]	P2: good *M* [CI]	P3: average *M* [CI]	P4: low-insecure *M* [CI]	Significant test
Life Satisfaction	4.269 [4.078; 4.443]	3.866 [3.645; 4.033]	3.850 [3.765; 3.958]	2.942 [2.816; 3.083]	P1 > P2 = P3 > P4
Self-kindness	3.009 [2.740; 3.279]	2.943 [2.743; 3.143]	3.083 [2.977; 3.189]	2.638 [2.531; 2.746]	P4 < P2 = P3 = P1
Self-judgement	1.666 [1.524; 1.785]	1.881 [1.757; 1.985]	2.369 [2.275; 2.448]	3.125[3.002; 3.229]	P1 < P2 < P3 < P4

*Note*. ***M***: mean value. CI: 95% confidence interval of mean value

## Discussion

Despite the wealth of research on global SE as well as its different facets (for an overview, [11]), few studies have jointly considered all three SE facets in childhood and adolescence or these facets’ relationship with PACR, self-kindness, self-judgement, and life satisfaction. This study, which employed a longitudinal, person-oriented approach, obtained four central results: (1) the identification of four SE profiles, (2) the confirmation of their high stability (particularly in relation to *low-insecure* SE), (3) evidence of the overall negative impact of PACR on SE development (promoting low, unstable, and highly contingent SE), and (4) a demonstration of the positive association between *optimal-secure* SE and less self-judgement, more self-kindness, and greater life satisfaction.

### Self-esteem profiles and their relationships

Four SE profiles could be identified based on the facets of SE level, stability, and contingency. In accordance with our hypothesis, the profiles *optimal-secure*, *average*, and *low-insecure* were detected in our sample. However, the profiles *high-insecure* and *learned helplessness* (low, stable, not contingent) were not detected.

Nonetheless, corresponding SE profiles are suspected to exist in the general population, as, for example, high-insecure SE has been extensively studied in the context of variable-oriented approaches. However, a corresponding SE profile may be more likely to be found in adults and particularly in clinical sub-populations, as it is primarily associated with narcissism (e.g., [28, 47, 50, 96]). Furthermore, an insecure SE profile with an average SE level as well as a profile of learned helplessness have been identified in a previous person-oriented approach, but only in a particularly large and selective sample [54]. Since learned helplessness is associated with a lack of perspective; prolonged failures; and internal, stable, and uncontrollable attributions (e.g., [97]), it can be assumed that a corresponding SE profile would develop primarily at older ages. Over the course of a child’s academic career, school becomes more evaluative and competitive, so that the evaluation of learning outcomes, as opposed to the learning process, becomes more of a focus [98]. At the same time, self-confidence regarding the ability to master school tasks decreases with age and experience [99], especially in the case of persistent negative experiences, which may favour the development of a low, stable, and non-contingent SE profile in the long run. Furthermore, it is conceivable that SE profiles of learned helplessness would be more prevalent in cultures with either a lack of perspective or even more controlling and failure-oriented parenting behaviours. For example, Chinese (vs. European American) children report that their parents emphasise their academic failures instead of their achievements [100]. Moreover, Chinese parents on average react more harshly than European American parents to their children’s failures, but at the same time show no positive reaction to their successes [101]. However, further research on SE profiles in different age and life domains, as well as across different cultures, is needed to better understand the consistency of the profiles and the conditions of their development.

Independently of the profiles that were expected but not found, another profile appeared that was not expected. In addition to the *optimal-secure* profile, which was only marginally represented (between 5% and 7% of the sample), another profile was identified in the upper SE range. The *good* profile showed a slightly lower SE level, slightly lower stability, and slightly higher contingency than the *optimal-secure* profile and was found in many more children and adolescents. Empirically, Crocker and Wolfe [102] found support for this finding and argued that there are hardly any people who show a non-contingent SE. Crocker [103] was able to demonstrate that children and adolescents rarely have a low score on the contingency domain of SE (< 5%).

This finding can also be explained in relation to the strong negative influence of parental conditional regard on SE development, which, in alignment with our hypothesis, we also found for both PANCR and PAPCR. For example, one study found that over 80% of parents use some kind of psychological control in the context of parenting [104], which then negatively affects SE development. Moreover, it is not only parents who significantly influence children’s SE development but also peers [105] and teachers [106]. Through direct or indirect feedback, such as the possible promotion of social comparisons or even positive conditional regard for good performance, peers and teachers can negatively impact the holistic development of SE. Cohen and colleagues [106] support this reasoning by showing that conditional regard is a widely used motivational strategy in the classroom, where it also frustrates children’s needs for autonomy and relatedness. However, further empirical data on how multiple relationships affect SE are lacking, which represents another interesting area of research [107]. Overall, this also provides theoretical support for the differentiation between *optimal-secure* SE (which develops only under truly optimal socialisation conditions at all levels and is therefore poorly represented) and *good* SE, which develops much more frequently in the general population.

At the same time, these theoretical and empirical explanations provide a possible explanation for the high number of children and adolescents with *low-insecure* SE and for the strong stability of this profile, regardless of the generally high profile stabilities and the possibility that they were overestimated due to the short measurement interval and thus stable contextual factors. In addition, it can be assumed that parental conditional regard has a double negative impact on children and adolescents, as it not only affects them directly but also undermines the development of their socioemotional competencies [40]. Therefore, social relationships are indirectly negatively influenced, decreasing general well-being and presumably further stabilising *low-insecure* SE. A similar effect is produced by the tendency, among children and adolescents whose parents use conditional regard, to experience their friends and partners as conditionally regarding [108].

The differentiated effects of PAPCR and PANCR on SE profiles could not be fully examined within the scope of this study. On the one hand, only minor qualitative differences emerged between the profiles; hence, it cannot be determined, for example, whether PAPCR is more likely to be related to high-insecure SE, while PANCR is more likely to be related to a profile of learned helplessness due to the additional negative influence on SE level (and not only on SE contingency; [67, 68]). However, the finding that use of PANCR yields an eightfold increase in the probability of belonging to the *low-insecure* compared to the *optimal-secure* SE profile is consistent with previous assumptions. This is especially true when considering that the probability of belonging to the *low-insecure* compared to the *optimal-secure* SE profile is only 1.5 times higher when PAPCR is used. On the other hand, this study did not identify whether PAPCR and PANCR co-occur to high, low, or varying degrees, but this point is important in relation to PACR’s association with SE (cf., [12]). Therefore, further research is needed to clarify the relationship between PAPCR, PANCR, and qualitatively different SE profiles.

Empirical findings and theoretical considerations (e.g., SDT; [57, 58]) regarding the negative relationship between PACR and SE are consistent with the results of this study. However, assumptions regarding a causal relationship between corresponding constructs should be treated with caution, as Otterpohl and colleagues [32] point out that it is child characteristics and behaviours, in the form of higher SE contingency, that influence parental conditional regard rather than the other way around. In order to avoid reopening the debate, regarding whether parents influence their children or vice versa [32], in the context of this study, it is important to interpret the corresponding findings correlatively. In line with well-known socialisation theories (e.g., *attachment theory* and *sociometer theory*), it is conceivable that parents influence their children’s SE via PACR. At the same time, there are also well-founded theoretical considerations that parents react with conditional regard to their children’s contingent SE [32] or use PACR because their own SE is contingent on their children’s performance [109]. Consequently, it can be concluded that increased PACR is related to *low-insecure* rather than *optimal-secure* SE.

Finally, in line with our hypotheses, life satisfaction and self-compassion in the form of increased self-kindness and reduced self-judgement were shown to be significantly positively associated with *optimal-secure* SE and significantly negatively associated with *low-insecure* SE. In addition, we found clear differences between all SE profiles in the degree of self-judgement. Regarding children and adolescents’ self-kindness, there was a significant difference only between the *low-insecure* SE profile on the one hand and the *optimal-secure*, *good*, and *average* profiles on the other, where the latter three did not differ significantly. This effect could not be explained statistically – for example, by differences in the variance of the two constructs – so further research is needed to elucidate the corresponding effects. This is relevant for research on resilient and healthy personalities as well positive psychology, in order to identify which mechanisms reduce self-judgement among children and adolescents with *optimal-secure* SE compared to children and adolescents with *good* or *average* SE. Placing the findings of this study in the context of previous research, it might be assumed that children and adolescents with *low-insecure* SE are at particular risk for developing depressive symptoms (although causality remains unclear). Indeed, Stolow and colleagues [110] showed that positive forms of self-compassion, in the form of self-kindness, predict a reduction in depressive symptoms, whereas no significant increase in depressive symptoms was found for negative forms of self-compassion in the form of self-judgement. However, other studies have provided ample evidence of the central role of self-judgement in the development of, for example, differentiated personality disorders [111, 112]. In any case, further research is needed, although the present findings are in line with previous studies indicating that high self-compassion is associated with a secure, positive self-image in adolescence (e.g., [111]).

### Self-esteem profiles and their stability

This study’s identification of mainly quantitative rather than qualitative differences between the profiles (forming a continuum comprising *optimal-secure*, *good*, *average*, and *low-insecure* SE) may challenge the assumption of independent SE facets. Profiles with strong discrepancies between SE instability and contingency were not found in children and adolescents (nor in the group of psychology and education undergraduates studied by Kärchner & Schwinger; [54]). Although qualitatively different profiles, such as high-insecure SE or learned helplessness, are still expected to occur in other sub-populations, these profiles do not show discrepancies between SE instability and contingency either. Because SE contingency is defined as the extent to which SE fluctuates in response to self-relevant events (e.g., [113]), consonance with SE instability is expected. This expectation is consistent with the frequently reported moderate positive correlations between SE instability and contingency (e.g., [33]), which were also confirmed by our findings. Nevertheless, the distinctiveness and utility of the SE facets of instability and contingency are evidenced by their weak correlation with SE level [114] and supported by the fact that they predict behaviour (e.g., aggression: [115]; verbal defensiveness: [116]) and psychological adjustment (e.g., depression: [117, 118]) beyond the effect of SE level. Thus, it should not be concluded from the results of this study that qualitatively different SE profiles do not exist. Rather, the question of the existence of profiles that differ in the facets of instability and contingency remains unresolved and requires further research.

Further research is also needed regarding the stability of SE profiles. This study found high profile stability over a period of six weeks, in accordance with our hypothesis, and transitions occurred predominantly to related profiles. Most profile transitions (in both directions) occurred between the profiles *optimal-secure* and *good*, which might reflect (the lack of) the reliability of the measurement instrument or the similarity of the two profiles. However, the *low-insecure* profile showed concerningly high stability, which might be due to self-fulfilling prophecies. Low, unstable, and highly contingent SE is associated with more self-handicapping, lower life satisfaction [54], social withdrawal, and poor psychosocial adjustment in general (e.g., [23, 25, 29]). Further research is urgently needed to test whether this high stability is also found in other samples and over longer time periods. If this is the case, research should aim to elucidate what this stability means for the long-term development of the children and adolescents concerned.

In contrast to the results of this study, when examining results on the development of the individual SE facets over the life span, we find that contingency and instability of SE develop similarly and decrease with age, whereas SE level increases with age [119]. These findings suggest that older people show more self-acceptance (e.g., [120]) and, as age increases, mistakes and failures have less impact on SE [119]. Another hypothesis comes from Crocker and Wolfe [102], who state that as people age, their SE becomes less dependent on external contingencies (e.g., praise and recognition from others) and gradually shifts to dependence on more internal contingencies (e.g., virtue). Thus, the SE of older people would fluctuate less due to external influences in everyday life (such as social feedback), and perceived SE stability would increase. The reduction of SE instability and contingency with increased age is further supported by the idea that older individuals exhibit more adaptive emotion regulation strategies, mindfulness, and acceptance (for further discussion see, [121]). They also have better daily routines [122] and tend to withdraw from negative interpersonal relationships [123].

Thus, when the different facets are considered independently, the findings suggest that SE develops positively over the life course, becoming higher, more stable, and less contingent. However, the question of how the combination of SE facets develops over the lifespan remains unanswered. Would the development trend be similarly positive? Based on the findings of this study, this does not seem to be the case.

### Limitations and future research

This study has some limitations that contribute to the formulation of new research questions. In our sample, we found predominantly quantitative differences in terms of profile compositions. Further studies should focus on larger samples as well as different sub-populations (e.g., different cultures, clinical samples) to identify qualitative profile differences and investigate their stability in the long run. This consideration leads to the second point of criticism: the six-week time interval is problematic due to the stability of contextual factors and may have led to an overestimation of profile stability. Further research should examine longer time intervals in childhood, adolescence, and adulthood and thereby map the influence of different developmental stages and significant environmental changes on the development of SE profiles. Another point of criticism arises from the fact that all the data on all the constructs in this study were collected from the perspectives of the children and adolescents, which may have led to an overestimation of effects due to common method variance. Thus, in future research, it would be interesting to collect data on parenting behaviours from the parents’ perspective, as it has repeatedly been demonstrated that self-perceptions and other-perceptions can differ significantly [124, 125]. In this context, the influence of parental conditional regard on global SE could be considered not only for different combinations of PAPCR and PANCR (cf., [12]) but also in relation to other domains, because parental conditional regard is usually measured domain-specifically [15] and was captured here only for the academic domain. Effects in different domains exert influence in the same direction, as they follow the same principle of frustration of basic psychological needs and introjection of shame and guilt, albeit with different effect sizes (see [15]). A similar limitation arises with respect to self-compassion, which was operationalised by only two of the original six facets. In further research, it would also be interesting to record and control for students’ grades, as the significant influence of academic success (e.g., in the form of grades) on the relationship between PACR and different SE facets has been demonstrated (e.g., [126]) but was not considered here. Last, as indicated above, it will be of central importance to consider not only parents but also peers and teachers as sources of conditional regard and to develop prevention and intervention studies with the aim of fostering positive changes towards a globally improved SE.

### Practical implications

Despite some limitations, the results of this study have significant practical implications. The high number of children and adolescents with a *low-insecure* SE and the high stability of this profile highlight the need for timely prevention to promote global SE, especially since numerous studies have already demonstrated the importance of SE in the aetiology of different psychological disorders [1, 3, 6]. Nevertheless, shifts to more positive profiles provide evidence that such a change is possible. This result underscores that interventions that aim to prevent mental disorders by promoting SE and averting far-reaching consequences are also central to children’s and adolescents’ well-being and life satisfaction. For example, one starting point would be to educate parents about the negative consequences of PANCR and PAPCR, as this study shows that PACR in general has a negative impact not only on the individual facets of SE but on global SE development.

## Conclusion

By using a longitudinal person-oriented approach, this study has made a significant contribution to elucidating the composition of *optimal-secure* SE and its relationship with parental conditional regard, self-kindness, self-judgement, and life satisfaction. *Optimal-secure* SE (high, stable, and not contingent) is associated with significantly more self-compassion and significantly higher life satisfaction and is thus a sign of a resilient personality, especially compared to *low-insecure* SE (low, unstable, and highly contingent). Given the important role of SE in the aetiology of mental disorders, more longitudinal, person-oriented studies should be conducted to identify particularly vulnerable SE profiles and the factors (in addition to parental conditional regard) that influence holistic SE development. Finally, person-centred approaches can help to clarify contradictory findings regarding optimal SE, which can be attributed to a lack of integration of the major SE facets.

### Electronic supplementary material

Below is the link to the electronic supplementary material.

Supplementary Material 1

## Data Availability

The datasets generated during and/or analysed during the current study are not publicly available [as there is no consent of the participants for the general publication of the data] but datasets are available from the corresponding author on reasonable request.

## List of Abbreviations

AIC: Akaike’s Information Criterion
ABIC: Adjusted Bayesian Information Criterion
BIC: Bayesian Information Criterion
CAIC: Consistent Akaike’s Information Criterion
CFA: Confirmatory Factor Analysis
LMR-LRT: Lo-Mendell-Rubin Likelihood Ratio Test
LPA: Latent Profile Analysis
LTA: Latent Transition Analysis
PANCR: Parental academic negative conditional regard
PAPCR: Parental academic positive conditional regard
PACR: Parental academic conditional regard
SDT: Self-Determination Theory
SE: Self-esteem
SES: Socioeconomic status

## References

[1] Kresznerits S, Rózsa S, Perczel-Forintos D (2022). A transdiagnostic model of low self-esteem: pathway analysis in a heterogeneous clinical sample. Behav Cogn Psychother.

[2] Zeigler-Hill V (2011). The connections between self-esteem and psychopathology. J Contemp Psychother.

[3] Sowislo JF, Orth U (2013). Does low self-esteem predict depression and anxiety? A meta-analysis of longitudinal studies. Psychol Bull.

[4] Beck AT (1967). Depression. Clinical, experimental and theoretical aspects.

[5] Abdel-Khalek AM, Holloway F (2016). Introduction to the psychology of self-esteem. Self-esteem: perspectives, influences and improvement strategies.

[6] Hilbert S, Goerigk S, Padberg F, Nadjiri A, Übleis A, Jobst A (2019). The role of self-esteem in Depression: a longitudinal study. Behav Cogn Psychother.

[7] Cvencek D, Greenwald AG, Meltzoff AN (2016). Implicit measures for preschool children confirm self-esteem’s role in maintaining a balanced identity. J Exp Soc Psychol.

[8] Krauss S, Orth U, Robins RW (2020). Family environment and self-esteem development: a longitudinal study from age 10 to 16. J Pers Soc Psychol.

[9] Brummelman E, Sedikides C (2020). Raising children with high self-esteem (but not narcissism). Child Dev Perspect.

[10] Rogers CR (1951). Client-centered therapy; its current practice, implications, and theory.

[11] Orth U, Robins RW (2022). Is high self-esteem beneficial? Revisiting a classic question. Am Psychol.

[12] Steffgen ST, Soenens B, Otterpohl N, Stiensmeier-Pelster J, Schwinger M (2022). Latent profiles of parental academic conditional positive and negative regard. Parenting.

[13] Baumeister RF, Campbell JD, Krueger JI, Vohs KD (2003). Does High Self-Esteem cause better performance, interpersonal success, happiness, or healthier Lifestyles?. Psychol Sci Public Interest.

[14] Twenge JM, Foster JD (2010). Birth cohort increases in narcissistic personality traits among American College Students, 1982–2009. Soc Psychol Personal Sci.

[15] Assor A, Roth G, Deci EL (2004). The emotional costs of parents’ conditional regard: a self-determination theory analysis. J Pers.

[16] Robins RW, Trzesniewski KH (2005). Self-Esteem Development across the Lifespan. Curr Dir Psychol Sci.

[17] Lucas RE, Donnellan MB (2011). Personality development across the life span: longitudinal analyses with a national sample from Germany. J Pers Soc Psychol.

[18] MacDonald G. Individual differences in self-esteem. In: Leary MR, Tangney JP, editors. Handbook of self and identity. The Guilford Press; 2012. pp. 354–77.

[19] Rosenberg M (1965). Society and the adolescent self-image.

[20] Diener E, Diener M, Diener E (2009). Cross-cultural correlates of life satisfaction and self-esteem. Culture and well-being.

[21] Bowles T (1999). Focusing on Time Orientation to explain adolescent self Concept and Academic Achievement: part II: testing a model. J Appl Health Behav.

[22] Rosenberg M, Schooler C, Schoenbach C, Rosenberg F (1995). Global self-esteem and specific Self-Esteem: different concepts, different outcomes. Am Sociol Rev.

[23] Zhou J, Li X, Tian L, Huebner ES (2020). Longitudinal association between low self-esteem and depression in early adolescents: the role of rejection sensitivity and loneliness. Psychol Psychother Theory Res Pract.

[24] Batool SS (2020). Academic achievement: interplay of positive parenting, self-esteem, and academic procrastination. Aust J Psychol.

[25] Masselink M, Van Roekel E, Oldehinkel AJ (2018). Self-esteem in early adolescence as predictor of depressive symptoms in late adolescence and early adulthood: the mediating role of motivational and social factors. J Youth Adolesc.

[26] Kernis MH, Cornell DP, Sun CR, Berry A, Harlow T (1993). There’s more to self-esteem than whether it is high or low: the importance of stability of self-esteem. J Pers Soc Psychol.

[27] Koszegi B, Loewenstein GF, Murooka T. Fragile self-esteem. SSRN Electron J. 2019.

[28] Kernis MH (2003). Toward a conceptualization of optimal self-esteem. Psychol Inq.

[29] Kernis MH, Grannemann BD, Mathis LC (1991). Stability of self-esteem as a moderator of the relation between level of self-esteem and depression. J Pers Soc Psychol.

[30] Kernis MH, Grannemann BD, Barclay LC (1989). Stability and level of self-esteem as predictors of anger arousal and hostility. J Pers Soc Psychol.

[31] Zeigler-Hill V, Enjaian B, Holden CJ, Southard AC (2014). Using self-esteem instability to disentangle the connection between self-esteem level and perceived aggression. J Res Personal.

[32] Otterpohl N, Bruch S, Stiensmeier-Pelster J, Steffgen T, Schöne C, Schwinger M (2021). Clarifying the connection between parental conditional regard and contingent self‐esteem: an examination of cross‐lagged relations in early adolescence. J Pers.

[33] Schöne C, Stiensmeier-Pelster J (2016). SEKJ - selbstwertinventar für Kinder und Jugendliche [Self-esteem inventory for children and adolescents].

[34] Sargent JT, Crocker J, Luhtanen RK (2006). Contingencies of self–worth and depressive symptoms in College Students. J Soc Clin Psychol.

[35] Schöne C, Tandler SS, Stiensmeier-Pelster J (2015). Contingent self-esteem and vulnerability to depression: academic contingent self-esteem predicts depressive symptoms in students. Front Psychol.

[36] Sheldon KM, Elliot AJ (1999). Goal striving, need satisfaction, and longitudinal well-being: the self-concordance model. J Pers Soc Psychol.

[37] Wouters S, Doumen S, Germeijs V, Colpin H, Verschueren K (2013). Contingencies of self-worth in early adolescence: the Antecedent Role of Perceived Parenting: parenting and contingencies of self-worth. Soc Dev.

[38] McCormick WH, Turner LA, Foster JD (2015). A model of perceived parenting, authenticity, contingent self-worth and internalized aggression among college students. Personal Individ Differ.

[39] Neff KD, Self-Compassion (2011). Self-Esteem, and well-being. Soc Personal Psychol Compass.

[40] Assor A, Kanat-Maymon Y, Roth G, Weinstein N (2014). Parental conditional regard: psychological costs and antecedents. Human Motivation and Interpersonal Relationships.

[41] Ryan RM, Brown KW, Kernis MH (2006). What is optimal Self-Esteem? The cultivation and consequences of contingent vs. true self-esteem as viewed from the self-determination theory perspective. Self-esteem issues and answers: a sourcebook of current perspectives.

[42] Ryan RM. Agency and organization: Intrinsic motivation, autonomy, and the self in psychological development. In: Nebraska Symposium on Motivation, 1992: Developmental perspectives on motivation., Lincoln. NE, US: University of Nebraska Press; 1993. p. 1–56. (Current theory and research in motivation, Vol. 40). 1340519

[43] Ryan RM, Deci EL, Grolnick WS, Cicchetti D, Cohen DJ (1995). Autonomy, relatedness, and the self: their relation to development and psychopathology. Developmental psychopathology.

[44] Koole SL, Kuhl J (2003). Search of the real self: a functional perspective on optimal self-esteem and authenticity. Psychol Inq.

[45] Rhodewalt F. Possessing and striving for high self-esteem. In: Kernis MH, editor. Self-esteem issues and answers: a sourcebook of current perspectives. Psychology Press; 2013. pp. 281–7.

[46] Rhodewalt F, Tragakis MW, Finnerty J (2006). Narcissism and self-handicapping: linking self-aggrandizement to behavior. J Res Personal.

[47] Tracy JL, Robins RW (2003). Death of a (narcissistic) salesman: an integrative model of Fragile Self-Esteem. Psychol Inq.

[48] Schröder-Abé M, Rudolph A, Schütz A (2007). High implicit self‐esteem is not necessarily advantageous: discrepancies between explicit and implicit self‐esteem and their relationship with anger expression and psychological health. Eur J Personal.

[49] Vater A, Ritter K, Schröder-Abé M, Schütz A, Lammers CH, Bosson JK (2013). When grandiosity and vulnerability collide: implicit and explicit self-esteem in patients with narcissistic personality disorder. J Behav Ther Exp Psychiatry.

[50] Zeigler-Hill V, Besser A, King K (2011). Contingent self-esteem and anticipated reactions to interpersonal rejection and achievement failure. J Soc Clin Psychol.

[51] Maier SF, Seligman ME (1976). Learned helplessness: theory and evidence. J Exp Psychol Gen.

[52] Dweck CS, Leggett EL (1988). A social-cognitive approach to motivation and personality. Psychol Rev.

[53] Perrone L, Borelli JL, Smiley P, Rasmussen HF, Hilt LM (2016). Do children’s attributions mediate the link between parental conditional regard and child depression and emotion?. J Child Fam Stud.

[54] Kärchner H, Schwinger M (2018). Selbstwertprofile und ihre Korrelate im Lern- und Leistungskontext: Eine latente profilanalyse [Self-esteem profiles and their correlates in the context of learning and achievement: a latent profile analysis]. Z Für Pädagog [Psychol Ger J Educ Psychol].

[55] Brown JD, Suls J (1993). Self-esteem and self-evaluation: feeling is believing. The self in social perspective.

[56] Trzesniewski KH, Donnellan MB, Robins RW (2003). Stability of self-esteem across the life span. J Pers Soc Psychol.

[57] Deci EL, Ryan RM, Kernis MH (1995). Human autonomy. Efficacy, Agency, and self-esteem.

[58] Deci EL, Ryan RM (2000). The what and why of goal pursuits: human needs and the self-determination of Behavior. Psychol Inq.

[59] Assor A, Tal K (2012). When parents’ affection depends on child’s achievement: parental conditional positive regard, self-aggrandizement, shame and coping in adolescents. J Adolesc.

[60] Otterpohl N, Steffgen ST, Stiensmeier-Pelster J, Brenning K, Soenens B (2020). The intergenerational continuity of parental conditional regard and its role in mothers’ and adolescents’ contingent self‐esteem and depressive symptoms. Soc Dev.

[61] Roth G, Assor A, Niemiec CP, Ryan RM, Deci EL (2009). The emotional and academic consequences of parental conditional regard: comparing conditional positive regard, conditional negative regard, and autonomy support as parenting practices. Dev Psychol.

[62] Assor A, Vansteenkiste M, Kaplan A (2009). Identified versus introjected approach and introjected avoidance motivations in school and in sports: the limited benefits of self-worth strivings. J Educ Psychol.

[63] Grolnick WS, Deci EL, Ryan RM, Grusec JE, Kuczynski L (1997). Internalization within the family. Parenting and children’s internalization of values: a handbook of contemporary theory.

[64] Curran T (2018). Parental conditional regard and the development of perfectionism in adolescent athletes: the mediating role of competence contingent self-worth. Sport Exerc Perform Psychol.

[65] Kollat SH. The role of conditional parental regard and excessively Contingent Self-Esteem in Children’s peer Relationships. Dissertation ed. Pennsylvania State University; 2007.

[66] Haines JE, Schutte NS (2023). Parental conditional regard: a meta-analysis. J Adolesc.

[67] Otterpohl N, Keil A, Assor A, Stiensmeier J (2017). Erfassung von elterlicher bedingter Wertschätzung im Lern- und Leistungsbereich und im Bereich der Emotionsregulation: Eine deutschsprachige Adaptation der parental conditional regard scale (PCR-D) [Assessment of parental conditional regard in learning, achievement, and emotion regulation: a german language adaptation of the parental conditional regard scale (PCR-D)]. Z Für Entwicklungspsychologie Pädagog. Psychol J Dev Educ Psychol.

[68] Wouters S, Colpin H, Luyckx K, Verschueren K (2018). Explaining the relationship between parenting and internalizing symptoms: the role of self-esteem level and contingency. J Child Fam Stud.

[69] Ryan RM, Deci EL (2017). Self-determination theory: basic psychological needs in motivation, development, and wellness.

[70] Neff KD (2003). The Development and Validation of a scale to measure Self-Compassion. Self Identity.

[71] Donald JN, Ciarrochi J, Parker PD, Sahdra BK, Marshall SL, Guo J (2018). A worthy self is a caring self: examining the developmental relations between self-esteem and self‐compassion in adolescents. J Pers.

[72] Fergusson DM, McLeod GFH, Horwood LJ, Swain NR, Chapple S, Poulton R (2015). Life satisfaction and mental health problems (18 to 35 years). Psychol Med.

[73] Benner AD, Boyle AE, Sadler S (2016). Parental involvement and adolescents’ Educational Success: the Roles of prior achievement and socioeconomic status. J Youth Adolesc.

[74] Hoff E, Laursen B. Socioeconomic status and parenting. Handbook of parenting. 3rd ed. Routledge; 2019.

[75] Klipker K, Baumgarten F, Göbel K, Lampert T, Hölling H (2018). Mental health problems in children and adolescents in Germany. Results of the cross-sectional KiGGS Wave 2 study and trends. J Health Monit.

[76] Orth U, Erol RY, Luciano EC (2018). Development of self-esteem from age 4 to 94 years: a meta-analysis of longitudinal studies. Psychol Bull.

[77] Ullman C, Tatar M (2001). Psychological Adjustment among israeli adolescent immigrants: a report on life satisfaction, Self-Concept, and self-esteem. J Youth Adolesc.

[78] Altinyelken HK (2009). Migration and self-esteem: a qualitative study among internal migrant girls in Turkey. Adolescence.

[79] Cohen J (1988). Statistical power analysis for the behavioral Sciences.

[80] Ellis PD (2010). The essential guide to Effect Sizes: statistical power, Meta-analysis, and the interpretation of Research results.

[81] Hayes AF, Coutts JJ (2020). Use Omega rather than Cronbach’s alpha for estimating reliability. But… Commun Methods Meas.

[82] McDonald RP. Test theory: a unified treatment. Psychology Press; 1999.

[83] Roth G (2008). Perceived parental conditional regard and autonomy support as predictors of young adults’ Self- Versus other-oriented prosocial tendencies. J Pers.

[84] Hupfeld J, Ruffieux N (2011). Validierung einer deutschen Version der Self-Compassion Scale (SCS-D) [Validation of a german version of the self-compassion scale (SCS-D)]. Z Für Klin Psychol psychother [Ger. J Clin Psychol Psychother].

[85] Diener E, Emmons RA, Larsen RJ, Griffin S (1985). The satisfaction with Life Scale. J Pers Assess.

[86] Pavot W, Diener E, Colvin CR, Sandvik E (1991). Further validation of the satisfaction with Life Scale: evidence for the Cross-Method Convergence of Well-Being measures. J Pers Assess.

[87] OECD. Students questionnaire of the OECD programme for international student assessment (PISA) 2009. Organisation for Economic Co-operation and Development; 2009.

[88] Morin AJS, Litalien D (2017). Longitudinal tests of profile similarity and latent transition analyses.

[89] Muthen LK, Muthen BO, Mplus. 8. 1998.

[90] Tang X, Wang MT, Parada F, Salmela-Aro K (2021). Putting the goal back into grit: academic goal commitment, grit, and academic achievement. J Youth Adolesc.

[91] Morin AJS, Litalien D. Mixture modeling for Lifespan Developmental Research. Oxford Research Encyclopedia of psychology. Oxford University Press; 2019.

[92] Gillet N, Morin AJS, Reeve J (2017). Stability, change, and implications of students’ motivation profiles: a latent transition analysis. Contemp Educ Psychol.

[93] Asparouhov T, Muthén B (2014). Auxiliary variables in mixture modeling: three-step approaches using M*plus*. Struct Equ Model Multidiscip J.

[94] Nylund KL, Asparouhov T, Muthén BO (2007). Deciding on the number of classes in latent class analysis and growth mixture modeling: a Monte Carlo Simulation Study. Struct Equ Model Multidiscip J.

[95] Spurk D, Hirschi A, Wang M, Valero D, Kauffeld S (2020). Latent profile analysis: a review and how to guide of its application within vocational behavior research. J Vocat Behav.

[96] Rhodewalt F. Possessing and striving for high self-esteem. Self-Esteem Issues Answ Sourceb Curr Perspect. 2006;281–7.

[97] Valås H (2001). Learned helplessness and Psychological Adjustment: Effects of age, gender and academic achievement. Scand J Educ Res.

[98] Eccles JS, Midgley C, Adler T (1984). Grade-related changes in the school environment: Effects on achievement motivation. Dev Achiev Motiv.

[99] Stipek DJ. Motivating students to learn: a lifelong perspective. National Commission on Excellence in Education; 1984.

[100] Ng FFY, Pomerantz EM, Lam Sfong (2007). European american and chinese parents’ responses to children’s success and failure: implications for children’s responses. Dev Psychol.

[101] Qin DB, Way N, Mukherjee P (2008). The other side of the model minority story: the familial and peer challenges faced by chinese american adolescents. Youth Soc.

[102] Crocker J, Wolfe CT (2001). Contingencies of self-worth. Psychol Rev.

[103] Crocker J (2002). Contingencies of Self-Worth: implications for self-regulation and psychological vulnerability. Self Identity.

[104] Teuber Z, Tang X, Sielemann L, Otterpohl N, Wild E (2022). Autonomy-related parenting profiles and their Effects on Adolescents’ academic and psychological development: a longitudinal person-oriented analysis. J Youth Adolesc.

[105] Gorrese A, Ruggieri R (2013). Peer attachment and self-esteem: a meta-analytic review. Personal Individ Differ.

[106] Cohen R, Moed A, Shoshani A, Roth G, Kanat-Maymon Y (2020). Teachers’ conditional regard and students’ need satisfaction and Agentic Engagement: a Multilevel Motivation Mediation Model. J Youth Adolesc.

[107] Verschueren K (2020). Attachment, self-esteem, and socio-emotional adjustment: there is more than just the mother. Attach Hum Dev.

[108] Moller AC, Roth G, Niemiec CP, Kanat-Maymon Y, Deci EL (2019). Mediators of the associations between parents’ conditional regard and the quality of their adult-children’s peer-relationships. Motiv Emot.

[109] Steffgen ST, Otterpohl N, Wessing F, Schwinger M, Assor A, Kanat-Maymon Y (2022). The process linking Child-Invested Contingent Self-Esteem and conditional regard: the roles of maternal anger and its regulation. J Child Fam Stud.

[110] Stolow D, Zuroff DC, Young JF, Karlin RA, Abela JRZ (2016). A prospective examination of Self-Compassion as a predictor of depressive symptoms in children and adolescents. J Soc Clin Psychol.

[111] Barry CT, Loflin DC, Doucette H (2015). Adolescent self-compassion: Associations with narcissism, self-esteem, aggression, and internalizing symptoms in at-risk males. Personal Individ Differ.

[112] Shin HS, Black DS, Shonkoff ET, Riggs NR, Pentz MA (2016). Associations among Dispositional Mindfulness, Self-Compassion, and executive function proficiency in early adolescents. Mindfulness.

[113] Crocker J, Park LE (2004). The costly pursuit of self-esteem. Psychol Bull.

[114] Okada R (2010). A meta-analytic review of the relation between self-esteem level and self-esteem instability. Personal Individ Differ.

[115] Webster GD, Kirkpatrick LA, Nezlek JB, Smith CV, Paddock EL (2007). Different slopes for different folks: self-esteem instability and gender as moderators of the relationship between self-esteem and attitudinal aggression. Self Identity.

[116] Kernis MH, Lakey CE, Heppner WL (2008). Secure versus fragile high self-esteem as a predictor of verbal defensiveness: converging findings across three different markers. J Pers.

[117] Cambron MJ, Acitelli LK, Steinberg L (2010). When friends make you blue: the role of friendship contingent self-esteem in predicting self-esteem and depressive symptoms. Pers Soc Psychol Bull.

[118] Franck E, De Raedt R (2007). Self-esteem reconsidered: unstable self-esteem outperforms level of self-esteem as vulnerability marker for depression. Behav Res Ther.

[119] Meier LL, Orth U, Denissen JJ, Kühnel A (2011). Age differences in instability, contingency, and level of self-esteem across the life span. J Res Personal.

[120] Ryff CD (1991). Possible selves in adulthood and old age: a tale of shifting horizons. Psychol Aging.

[121] Isaacowitz DM (2022). What do we know about aging and emotion regulation?. Perspect Psychol Sci J Assoc Psychol Sci.

[122] Bouisson J, Swendsen J (2003). Routinization and Emotional Well-Being: an experience Sampling Investigation in an Elderly French Sample. J Gerontol B Psychol Sci Soc Sci.

[123] Coats AH, Blanchard-Fields F (2008). Emotion regulation in interpersonal problems: the role of cognitive-emotional complexity, emotion regulation goals, and expressivity. Psychol Aging.

[124] Lohaus A, Vierhaus M (2014). Beurteilerdiskrepanzen bei der Erfassung internalisierenden/ externalisierenden verhaltens [Parent-Child discrepancies in the Assessment of Internalizing/Externalizing Behavior]. Z Für Entwicklungspsychologie Pädagog [Psychol J Dev Educ Psychol].

[125] Taber SM (2010). The veridicality of children’s reports of parenting: a review of factors contributing to parent–child discrepancies. Clin Psychol Rev.

[126] Brummelman E, Crocker J, Bushman BJ (2016). The praise Paradox: when and why praise backfires in Children with Low Self-Esteem. Child Dev Perspect.

