# Supplementary material for: What if parental love is conditional …? Children’s self-esteem profiles and their relationship with parental conditional regard and self-kindness
Source: BMC Psychol. 2023 Oct 9;11:322. doi: 10.1186/s40359-023-01380-3 (PMC10563255; doi:10.1186/s40359-023-01380-3)
Supplement: Supplementary file 1 — Supplementary Material 1 [file 40359_2023_1380_MOESM1_ESM.docx]

# Appendices

**Appendix 1**

|  | PANCR_t1 | PANCR_t2 | PAPCR_t1 | PAPCR_t2 | LSE_t1 | LSE_t2 | SSE_t1 | SSE_t2 | CSE_t1 | CSE_t2 | SK_t1 | SK_t2 | SJ_t1 | SJ_t2 | LS_t1 | LS_t2 |
| --- | --- | --- | --- | --- | --- | --- | --- | --- | --- | --- | --- | --- | --- | --- | --- | --- |
| PANCR_t1 | (1.37) | .689** | .427** | .419*** | -.234** | -.249** | -.172** | -.132** | .301** | .271** | -.145** | -.109** | .203** | .227** | -.267** | -.229** |
| PANCR_t2 |  | (1.41) | .365** | .537** | -.192** | -.267** | -.180*** | -.206** | .285** | .296** | -.131** | -.122** | .210** | .234** | -.232** | -.265** |
| PAPCR_t1 |  |  | (2.06) | .549** | -.016 | -.039 | -.085* | -.050 | .292** | .284** | -.083* | -.014 | .114* | .090* | -.047 | -.106* |
| PAPCR_t2 |  |  |  | (2.02) | -.101* | -.130** | -.134** | -.185** | .326** | .369** | -.088* | -.005 | .147** | .210** | -.161** | -.178** |
| LSE_t1 |  |  |  |  | (3.70) | .696** | .312** | .364** | -.251** | -.284** | .388** | .304** | -.391** | -.382** | .585** | .439** |
| LSE_t2 |  |  |  |  |  | (3.70) | .326** | .336** | -.229** | -.254** | .361** | .367** | -.398** | -.399** | .528** | .534** |
| SSE_t1 |  |  |  |  |  |  | (3.51) | .576** | -.451** | -.370** | .062 | .074 | -.431** | -.332** | .272** | .251** |
| SSE_t2 |  |  |  |  |  |  |  | (2.57) | -.348** | -.498** | .085* | .063 | -.380** | .431** | .301** | .274** |
| CSE_t1 |  |  |  |  |  |  |  |  | (2.62) | .641** | -.085* | -.063 | .421** | .392** | -.226** | -.168** |
| CSE_t2 |  |  |  |  |  |  |  |  |  | (2.53) | -.110** | -.061 | .378** | .484** | -.238** | -.151** |
| SK_t1 |  |  |  |  |  |  |  |  |  |  | (2.97) | .549** | -.132** | -.169** | .435** | .369** |
| SK_t2 |  |  |  |  |  |  |  |  |  |  |  | (2.80) | -.092* | -.082* | .286** | .396** |
| SJ_t1 |  |  |  |  |  |  |  |  |  |  |  |  | (2.53) | .640** | -.308** | -.276** |
| SJ_t2 |  |  |  |  |  |  |  |  |  |  |  |  |  | (2.45) | -.306** | -.269** |
| LS_t1 |  |  |  |  |  |  |  |  |  |  |  |  |  |  | (3.59) | .650** |
| LS_t2 |  |  |  |  |  |  |  |  |  |  |  |  |  |  |  | (3.52) |

*Scale Means (in Parantheses) and Zero-Order-Correlations between Variables Used in Present Study*

*Note.* PANCR = parental academic negative conditional regard. PAPCR = parental academic positive conditional regard. LSE = level of self-esteem. SSE = stability of self-esteem. CSE = contingency of self-esteem. SK = self-kindness. SJ = self-judgement. LS = life satisfaction. t1 = measurement time point 1. t2 = measurement time point 2. * *p* <. 05; ** *p* <.01.

**Appendix 2**

*Results from Confirmatory Factor Analysis Models for Self-Esteem Facets and Their Longitudinal Measurement Invariance*

| Model | χ2 | *df* | | *p* | CFI | | RMSEA | | 90% CI for RMSEA | SRMR |
| --- | --- | --- | --- | --- | --- | --- | --- | --- | --- | --- |
| *Cross-sectional confirmatory factor analysis analyses* | | | | | | |  | |  |  |
| T1 | 71.52 | 50 | | .025 | .982 | | .028 | | .011/.042 | .035 |
| T2 | 106.864 | 50 | | < .001 | .967 | | .044 | | .033/.055 | .040 |
| *Longitudinal measurement invariance* | | |  | | |  | |  |  |  |
| Configural MI | 616.51 | 225 | | < .001 | .925 | | .054 | | .049/.060 | .057 |
| Weak MI | 636.915 | 237 | | < .001 | .924 | | .054 | | .049/.059 | .060 |
| **Strong MI** | 683.647 | 249 | | < .001 | .917 | | .055 | | .050/.059 | .061 |

*Note.* *N* = 587.

**Appendix 3**

|  | #profiles | LL | #fp | Scaling | AIC | CAIC | BIC | ABIC | Entropy | LMR-LTR |
| --- | --- | --- | --- | --- | --- | --- | --- | --- | --- | --- |
| T1 | 1 | -2327.212 | 6 | 0.8794 | 4666.424 | 4698.674 | 4692.675 | 4673.627 | N/A |  |
|  | 2 | -2035.574 | 13 | 1.2231 | 4097.148 | 4167.023 | 4154.023 | 4112.753 | .873 | <.001 |
|  | 3 | -1952.441 | 20 | 1.1497 | 3944.881 | 4052.382 | 4032.382 | 3968.888 | .820 | .0013 |
|  | **4** | -1897.934 | 27 | 1.1883 | 3849.867 | 3994.994 | 3967.993 | 3882.278 | .778 | .0420 |
|  | 5 | -1877.812 | 34 | 1.1323 | 3823.625 | 4006.375 | 3972.375 | 3864.437 | .725 | .1164 |
|  | 6 | -1853.052 | 41 | 1.1098 | 3788.104 | 4008.480 | 3967.480 | 3837.320 | .749 | .1145 |
| T2 | 1 | -2393.282 | 6 | 0.8804 | 4798.564 | 4830.814 | 4824.815 | 4805.767 | N/A |  |
|  | 2 | -2093.885 | 13 | 1.6398 | 4213.770 | 4283.645 | 4270.645 | 4229.374 | .910 | .0136 |
|  | 3 | -1976.874 | 20 | 1.3547 | 3993.748 | 4101.248 | 4081.249 | 4017.756 | .815 | .0016 |
|  | **4** | -1925.928 | 27 | 1.3657 | 3905.857 | 4050.982 | 4023.983 | 3938.267 | .821 | .1905 |
|  | 5 | -1891.317 | 34 | 1.6068 | 3850.634 | 4033.385 | 3999.384 | 3891.446 | .778 | .6411 |
|  | 6 | -1850.685 | 41 | 1.2319 | 3783.371 | 4003.746 | 3962.747 | 3832.586 | .794 | .3643 |

*Results from Latent Profile Analysis Models Estimated Separately at Each Measurement Occasion*

*Note.* *N* = 587. #profiles = number of profiles. LL = model likelihood. #fp = number of free parameters. Scaling = scaling correction factor associated with robust maximum likelihood estimates. AIC = Akaike information criteria. CAIC = Consistent AIC. BIC = Bayesian information criteria. ABIC = sample size adjusted BIC. LMR-LTR = Lo-Mendell-Rubin adjusted LRT Test.

**Appendix 4**

*Results from Final Latent Profile Analysis Models and Latent Transition Analysis Models*

| Model | LL | #fp | Scaling | AIC | CAIC | BIC | ABIC | Entropy |
| --- | --- | --- | --- | --- | --- | --- | --- | --- |
| *Final Cross-Sectional Latent Profile Analyses* | | | | | | | | |
| T1 (4 profiles) | -1897.934 | 27 | 1.1883 | 3849.867 | 3994.994 | 3967.993 | 3882.278 | .778 |
| T2 (4 profiles)^ns^ | -1925.928 | 27 | 1.3657 | 3905.857 | 4050.982 | 4023.983 | 3938.267 | .821 |
| *Longitudinal Latent Profile Analyses* | | | | | | | | |
| Configural Similarity | -3823.862 | 54 | 1.2770 | 7755.724 | 8045.975 | 7991.976 | 7820.545 | .799 |
| Structural Similarity | -3844.734 | 42 | 1.2331 | 7773.468 | 7999.219 | 7957.219 | 7823.884 | .766 |
| Dispersion Similarity | -3856.300 | 30 | 1.9023 | 7772.601 | 7933.851 | 7903.851 | 7808.612 | .767 |
| Distributional Similarity | -3857.603 | 27 | 2.1871 | 7769.205 | 7914.332 | 7887.331 | 7801.615 | .768 |
| *Latent Transition Analysis* | -1244.534 | 15 | 0.7997 | 2519.067 | 2599,693 | 2584.693 | 2537.073 | .848 |

*Note.* ^ns^ Lo-Mendell-Rubin adjusted LRT Test was not significant. LL = model loglikelihood. #fp = number of free parameters. Scaling = scaling correction factor associated with robust maximum likelihood estimates. AIC = Akaike information criteria. CAIC = consistent AIC. BIC = Bayesian information criteria. ABIC = sample size adjusted BIC.
